# 962. Essential Consultants’ Skills and Attitudes (Willing CONSULT): A Cross-Sectional Survey

**DOI:** 10.1093/ofid/ofab466.1157

**Published:** 2021-12-04

**Authors:** Takahiro Matsuo, Kuniyoshi Hayashi, Yuki Uehara, Nobuyoshi Mori

**Affiliations:** 1 St. Luke’s International Hospital/The University of Texas Health Science Center at Houston, McGovern Medical School/The University of Texas MD Anderson Cancer Center, Houston, Texas; 2 St. Luke’s International University, Graduate School of Public Health, Tokyo, Tokyo, Japan; 3 St. Luke’s International Hospital, Tokyo, Tokyo, Japan

## Abstract

**Background:**

There is an increasing number of studies that infectious diseases consultations improve patients’ outcomes, but few studies have investigated the quality of consultations. The aim of this study was to identify important skills and attitudes for consultants to improve the quality of consultations.

**Methods:**

We conducted our research in two phases: a preliminary survey (May 1 to 14, 2020) and the main survey (June 1 to 14, 2020). As a preliminary survey, first-year postgraduate residents at St. Luke’s International Hospital in Tokyo, Japan, were first asked an open-ended question about the types of skills and attitudes that are important for consultants. After eliminating duplicate answers, there were 19 skills and attitudes in total. In the main survey with residents who completed their residency training at our institute, from 2014 to 2018, and current residents (2019–2020), we first asked them about their demographic characteristics (gender, years of postgraduate education, and type of specialty). Then, they answered how important each skill and attitude are for consultants. All 19 items were scored on a seven-point Likert scale that ranged from 0 (*completely disagree*) to 6 (*totally agree*) (Figure 1). Cronbach’s alpha confirmed the internal consistency of the questionnaire items. Principal component analysis and exploratory factor analysis were performed.

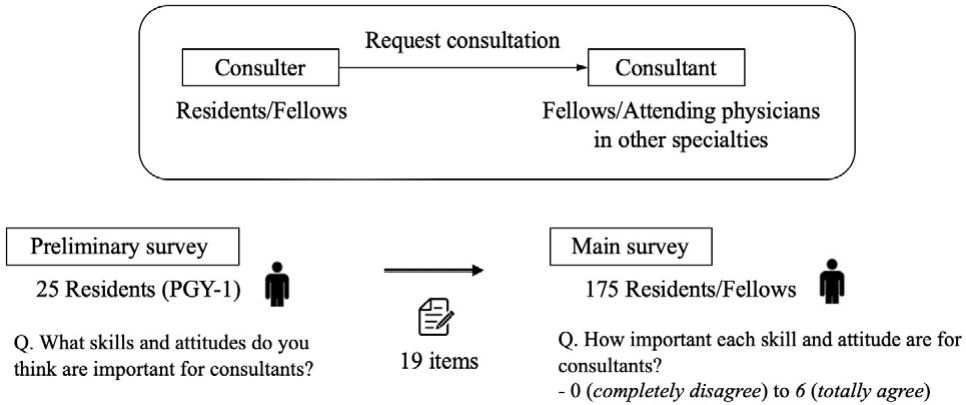

Figure 1. Skills and attitudes required in consultants according to residents

**Results:**

The survey included 107 individuals (61.1%, 175 potential participants). The median postgraduate years of education were four (interquartile range: 2-5), and 64.5% were men (*n* = 69). Seven key elements for consultants were identified and termed Willing CONSULT. These included (1) willingness (willingness to accept consultation requests), (2) contact (easy access to consultants), (3) needs (consideration of consulters’ needs), (4) suggestions and support (providing clear recommendations and suggestions, following up on the patients, and supporting the consulters continuously), (5) urgency (considering the situation’s urgency and responding appropriately), (6) learning opportunities (providing teaching points), and (7) text (writing medical records appropriately and quickly) (Figure 2).

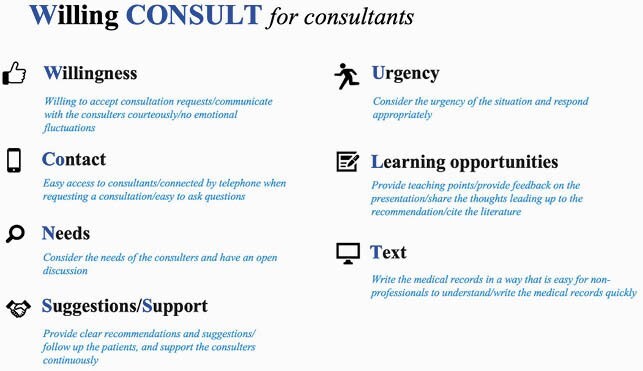

**Conclusion:**

We propose Willing CONSULT, which are important skills and attitudes for consultants.

**Disclosures:**

**All Authors**: No reported disclosures

